# Contribution of Rare Variants of the *SLC22A12* Gene to the Missing Heritability of Serum Urate Levels

**DOI:** 10.1534/genetics.119.303006

**Published:** 2020-01-31

**Authors:** Kazuharu Misawa, Takanori Hasegawa, Eikan Mishima, Promsuk Jutabha, Motoshi Ouchi, Kaname Kojima, Yosuke Kawai, Masafumi Matsuo, Naohiko Anzai, Masao Nagasaki

**Affiliations:** * Department of Genome Analysis, Institute of Biomedical Science, Kansai Medical University, 2-5-1 Shin-machi, Hirakata, Osaka 573-1010, Japan; † Tohoku Medical Megabank Organization, Tohoku University, Sendai, Miyagi 980-8573, Japan; ‡ RIKEN Center for Advanced Intelligence Project, 1-4-1 Nihonbashi, Chuo-ku, Tokyo 103-0027 Japan; § Health Intelligence Center, Institute of Medical Science, The University of Tokyo, Tokyo 108-8639, Japan; ** Division of Nephrology, Endocrinology and Vascular Medicine, Tohoku University Hospital, Sendai, Miyagi 980-8574, Japan; †† Chakri Naruebodindra Medical Institute, Faculty of Medicine Ramathibodi Hospital, Bang Phli, Samut Prakan 10540, Thailand; ‡‡ Department of Pharmacology and Toxicology, Dokkyo Medical University School of Medicine, Tochigi 321-0293, Japan; §§ National Center for Global Health and Medicine, Tokyo 162-8655, Japan; *** KNC Department of Nucleic Acid Drug Discovery, Faculty of Rehabilitation, Kobe Gakuin University, Nishi, Kobe 651-2180, Japan; ††† Research Center for Locomotion Biology, Kobe Gakuin University, Nishi, Kobe, 651-2180, Japan; ‡‡‡ Department of Pharmacology, Chiba University Graduate School of Medicine, Chiba 260-8670, Japan; §§§ Center for Genomic Medicine, Kyoto University Graduate School of Medicine, Shogoinkawaramachi 53, Sakyo-ku, Kyoto 606-8507, Japan; **** Human Biosciences Unit for the Top Global Course, Center for the Promotion of Interdisciplinary Education and Research (CPIER), Kyoto University, Shogoinkawaramachi 53, Sakyo-ku, Kyoto 606-8507, Japan

**Keywords:** serum uric acids, heritability, rare variants, transporter, metabolic syndrome, genetic factors, environmental factors, cohort study

## Abstract

Gout is a common arthritis caused by monosodium urate crystals. The heritability of serum urate levels is estimated to be 30–70%; however, common genetic variants account for only 7.9% of the variance in serum urate levels. This discrepancy is an example of “missing heritability.” The “missing heritability” suggests that variants associated with uric acid levels are yet to be found. By using genomic sequences of the ToMMo cohort, we identified rare variants of the *SLC22A12* gene that affect the urate transport activity of URAT1. URAT1 is a transporter protein encoded by the *SLC22A12* gene. We grouped the participants with variants affecting urate uptake by URAT1 and analyzed the variance of serum urate levels. The results showed that the heritability explained by the *SLC22A12* variants of men and women exceeds 10%, suggesting that rare variants underlie a substantial portion of the “missing heritability” of serum urate levels.

GOUT is one of the most common types of inflammatory arthritis caused by hyperuricemia. The balance between production of urate and urate excretion pathways determines an individual’s serum urate levels (Dalbeth *et al.* 2016). This balance can be modified by both genetic and environmental factors. Heritability estimates of serum urate span a range of 30–70% (Whitfield and Martin 1983; Emmerson *et al.* 1992; Yang *et al.* 2005; Nath *et al.* 2007; Vitart *et al.* 2008; MacCluer *et al.* 2010; Krishnan *et al.* 2012; Wang *et al.* 2018).

Serum urate significantly associates with 30 separate genetic loci as reported by a genome-wide association study (GWAS) of >16,000 European individuals (Köttgen *et al.* 2013). A weighted serum-urate genetic risk score constructed by using these variants accounted for 7.9% of the variance (Major *et al.* 2018b). Recently, Nakatochi *et al.* (2019) estimated the single nucleotide polymorphism (SNP)-based heritability (denoted *h*^2^_SNP_) of serum urate level in a Japanese meta-analysis and in European individuals (Köttgen *et al.* 2013) by using linkage disequilibrium (LD) score regression (Bulik-Sullivan *et al.* 2015). The heritability estimates were calculated from summary statistics of 1,447,573 SNPs, which were assessed in both studies, and have minor allele frequencies ≥1% in both studies. The *h*^2^_SNP_ ± sampling error (SE) estimates were 14.0% ± 4.3% for the Japanese study and 14.4% ± 3.9% for the European study. In other words, the estimated proportion of the variance explained by genome-wide significant SNPs discovered by GWAS (denoted *h*^2^_GWA_ ) and by LD score regression was only a fraction of the heritability estimated by family or twin studies. This is known as the “missing heritability” problem (Manolio *et al.* 2009). Missing heritability of serum urate levels indicates that as yet undiscovered variants might contribute to the phenotypic variations.

We hypothesized that rare functional SNPs are contributors to the missing heritability of serum urate levels. A previous study showed that the minor allele frequency (MAF) distribution of damaging SNPs was shifted toward rare SNPs compared with the MAF distribution of synonymous SNPs that are not likely to be functional (Gorlov *et al.* 2008).

In this study, we focused on the *SLC22A12* gene. This gene encodes a transporter protein known as URAT1. URAT1 has been identified as a urate-anion exchanger that affects serum urate level via urate reabsorption in human kidneys (Merriman 2015; Major *et al.* 2018a). It was shown that *SLC22A12* mutations lower the serum urate level (Enomoto *et al.* 2002; Ichida *et al.* 2004; Iwai *et al.* 2004; Mancikova *et al.* 2016). Variants of *SLC22A12* were reported in the European American, African American (Tin *et al.* 2018), and Czech populations (Stiburkova *et al.* 2013, 2015; Mancikova *et al.* 2016), as well as German populations of European ancestry (Graessler *et al.* 2006), Japanese (Ichida *et al.* 2004; Sakiyama *et al.* 2016), and Korean populations (Lee *et al.* 2008; Cho *et al.* 2015), along with a subgroup of the Roma population from five regions in three European countries (Slovakia, Czech Republic, and Spain) (Claverie-Martin *et al.* 2018), and Sri Lanka (Vidanapathirana *et al.* 2018). In this study, we searched for both common and rare variations of *SLC22A12* using whole-genome sequences of cohort participants of the Tohoku Medical Megabank project (TMM) conducted in the northern part of Japan (Kuriyama *et al.* 2016). We identified new variants and carried out experiments to examine whether they affect the resulting protein variants. Then, we carried out a functional analysis to test whether amino acid substitutions actively change the urate transporter activity without altering protein expression or membrane translocation of URAT1. We also accounted for the loss-of-function mechanism of missense mutations in URAT1 by exon skipping.

Several studies explored the link between increased serum urate levels and various components of metabolic syndrome, such as body mass index (BMI), diabetes, and glucose levels (Cook *et al.* 1986; Choi *et al.* 2005; Choi and Ford 2008; Stibůrkova *et al.* 2014; Davies *et al.* 2015; van Bommel *et al.* 2017; Ouchi *et al.* 2018). Multivariable modeling was performed after adjusting for potential covariates: age at examination, estimated glomerular filtration rate (eGFR), BMI, and hemoglobin A1c (HbA1c).

## Materials and Methods

### Sample information

The URAT1 protein is coded by *SLC22A12* and has an amino acid length of 553. We used the whole-genome sequences (WGS) of participants of the TMM cohort study to identify the *SLC22A12* variants. The TMM project was conducted by the Tohoku University Tohoku Medical Megabank Organization (ToMMo) and the Iwate Medical University Iwate Tohoku Medical Megabank Organization (IMM) (Kuriyama *et al.* 2016). A total of 3392 candidates were selected considering the traceability of participant information, and the quality and abundance of DNA samples for SNP array genotyping and WGS analysis. All candidates were genotyped with Illumina HumanOmni2.5-8 BeadChip (Omni2.5). From the candidates, 2306 samples were selected by filtering out close relatives of individual subjects, based on mean identity-by-descent (IBD; PIHAT in PLINK version 1.07) values indicating relatedness closer than third-degree relatives.

### Whole-genome sequences

Samples were handled in 96-well plates during library construction. Genomic DNA samples were diluted to a concentration of 20 ng/µl using a laboratory automation system (Biomek NXP, Beckman), and fragmented using a 96-well plate DNA sonication system (Covaris LE220, Covaris) to an average target size of 550 bp. The sheared DNA was subjected to library construction with the TruSeq DNA PCR-Free HT sample prep kit (Illumina) using a Bravo liquid-handling instrument (Agilent Technologies). Finally, the completed libraries were transferred to 1.5-ml tubes labeled with a barcode, and were then denatured or neutralized, and processed for library quality control (QC).

Library quantitation and QC were performed with quantitative MiSeq (qMiSeq) (Katsuoka *et al.* 2014). In this protocol, 8 or 10 µl of prepared libraries was denatured with an equal volume of 0.1 *N* NaOH for 5 min at room temperature and diluted with a 49-fold volume of ice-cold Illumina HT1 buffer. We pooled 50 µl of 96 denatured libraries including three control samples (those examined earlier on HiSeq). Then, 60 µl of the pooled library was diluted with 540 µl of ice-cold Illumina HT1 buffer, and analyzed with MiSeq using a 25-bp, paired-end protocol. The index ratio determined by the MiSeq sequencer was used as a relative concentration to determine the condition for the HiSeq run. Additionally, an electrophoretic analysis using the Fragment Analyzer (Advanced Analytical) software was performed as part of the library QC.

DNA libraries were analyzed using the HiSeq 2500 sequencing system, according to the manufacturer’s protocol. A TruSeq Rapid PE Cluster Kit (Illumina), and one-and-a-half TruSeq Rapid SBS Kit (200 cycles, Illumina), were used to perform a 162 bp, paired-end read in Rapid-Run Mode. Based on the qMiSeq results, the libraries were diluted to the appropriate concentrations, and used for on-board cluster generation (Illumina). We routinely checked the cluster density at the first base report, and decided whether to continue the analysis depending on the density (∼550–650 K/mm^2^).

The sequence reads from each sample were aligned to the reference human genome (GRCh37/hg19) with the decoy sequence (hs37d5). Based on previous studies (Li 2014; Nagasaki *et al.* 2015), Bowtie2 (version 2.1.0) with the “-X 2000” option was used for mapping. The bcftools software (ver. 0.1.17-dev) and the Genome Analysis Toolkit (GATK version 2.5-2) were applied to each aligned result, respectively, to call the single‐nucleotide variants (SNVs). For each sample, the read depth of each SNV position was calculated for the downstream filtering steps. Here, the read depth represents the total number of sequence reads aligned to the SNV position, with the mapping quality being ≥5. The SNVs were filtered out when read depths were extraordinarily low or high to avoid repetitive sequence of the genome. The SNVs were grouped by read depth of next generation sequencing (NGS). We then filtered out the SNV loci, in which >10% of the samples were not genotyped due to the depth filter applied in the previous step.

In total, the whole genome sequences of 2049 individuals were obtained. We call this dataset 2KJPN. See details on the website of the integrative Japanese Genome Variation Database (Yamaguchi-Kabata *et al.* 2015).

### Filtering participants

We excluded women who had undergone ovariectomy, because serum urate levels might be affected by reproductive hormones (Mumford *et al.* 2013). We also excluded individuals who were taking medicines, including losartan, allopurinol, febuxostat, benzbromarone, probenecid, bucolome, and topiroxostat, which reduce urate levels.

According to the questionnaire included in the ToMMo cohort study, ∼40% of diabetes patients in the ToMMo attended hospital regularly. About 28% and 29% of the cohort reported self-management of their lifestyle, with and without the guidance of medical doctors, respectively. About 3% dropped out from diabetic care. The questionnaire did not capture information on the start and end date of medication; therefore, a qualitative evaluation of diabetes treatment was difficult in this study. Thus, we excluded subjects who were diagnosed with diabetes. Instead, we used HbA1c as a covariate of serum urate level of participants without diabetes to examine the effect of glucose levels.

Among 2049 individuals, 771 were subsequently excluded. Of these, 11 withdrew their consent to participate, 585 subjects had missing data, 48 women had undergone ovarian surgery, 75 subjects were diagnosed with diabetes, and 85 were taking urate-lowering drugs. The excluded participants overlapped. The remaining 1285 participants were investigated in this study. We analyzed samples from men and women separately.

### Detecting URAT1 expression in *Xenopus* oocytes by immunofluorescence

The QuikChange II site-directed mutagenesis kit (Agilent Technologies) was used to introduce point mutations into the URAT1-pcDNA3.1 plasmid according to the manufacturer’s instructions. DNA sequencing was performed to confirm the cDNA sequences. Wild-type and mutant complementary RNAs (cRNAs) were synthesized by *in vitro* transcription, using a T7 mMESSAGE mMACHINE cRNA synthesis kit (Ambion).

Defolliculated *Xenopus* oocytes (stage IV–V) were injected with 20 ng of *URAT1* cRNA, and incubated in modified Barth’s solution [88 mM NaCl, 1 mM KCl, 0.33 mM Ca(NO_3_)_2_, 0.4 mM CaCl_2_, 0.8 mM MgSO_4_, 2.4 mM NaHCO_3_, and 10 mM HEPES, pH 7.4] containing gentamicin (50 μg/mL); oocytes were incubated at 18° for 2 or 3 days.

For immunofluorescence, cRNA-injected *Xenopus* oocytes were fixed with paraformaldehyde, embedded in paraffin blocks, and sectioned. Sections were incubated with anti-URAT1 polyclonal antibody (0.8 µg/ml, Atlas Antibodies) at 4° overnight, followed by Alexa568 at room temperature for 1 hr. Sections were examined using a confocal laser-scanning microscope.

Urate transport activity was examined after making the following amino acid substitutions in URAT1: p.Leu98Phe, p.Ala209Val, p.Ala226Val, p.Ala227Thr, p.Gln297Ter, p.Lys308Arg, p.Val469Ala, p.Gln533Lys, and p.His540Tyr. Uptake of urate (10 µM) was measured in 10 oocytes injected with wild-type or variant cRNAs.

### Expression of functional *URAT1* variants

Uptake experiments were performed at room temperature for 60 min in uptake buffer (96 mM sodium gluconate, 2 mM potassium gluconate, 1.8 mM calcium gluconate, 1 mM MgSO_4_, and 5 mM HEPES, pH 7.4) containing 10 µM [^14^C]urate (Moravek) to determine URAT1 transport activity. Oocytes were washed five times with ice-cold uptake solution and solubilized with 5% SDS. Radioactivity was measured in each oocyte by scintillation counting. Urate uptake into water-injected oocytes was used as the negative control. Differences of uptake between wildtype and variants were tested by Welch two-sample *t*-test.

### Splicing

*In vitro* splicing assays, using a hybrid minigene approach, were performed as described previously (Takeuchi *et al.* 2015). Minigene constructs were generated using the H492 vector (Habara *et al.* 2008). The target exons of human *SLC22A12* (*hSLC22A12*), including ∼150 nucleotides flanking shortened introns with *Nhe*I and *Bam*HI restriction sites, were amplified from the purchased human genome (Promega) using the following primers: forward for intron 1, 5′-aaagctagcagcctcctcctctcccatc-3′; reverse for intron 2, 5′-aaaggatccccagcaagtagggcgctttc-3′; forward for intron 2, 5′-aaagctagctgtaggtttcacccaggtgc-3′; reverse for intron 3, 5′-aaaggatccagctgaagcccagagagttc-3′. Both edges of the shortened introns were appropriately designed by the Human Splicing Finder program (http://www.umd.be/HSF/) to avoid activation of cryptic splicing. Each amplified fragment was inserted into the H492 vector, and targeted mutations were generated by site-directed mutagenesis. For the *in vitro* splicing assay, HEK293T cells obtained from the American Type Culture Collection were grown in Dulbecco’s modified Eagle’s medium with 10% fetal bovine serum, 100 units/ml penicillin, and 100 μg/ml streptomycin. After spreading the cells in six-well plates, the plasmids were transfected with Lipofectamine 2000 (Invitrogen). At 36 hr after transfection, total cellular RNA was extracted using RNeasy Mini Kit (Qiagen) and transcribed using ReverTra Ace qPCR RT Master Mix with gDNA Remover (Toyobo). PCR was performed using a forward primer corresponding to a segment upstream of exon A and a reverse primer complementary to a segment downstream of exon B: forward, 5′-acagctggattactcgctca-3′; reverse 5′-cagccagttaagtctctcac-3′.

### Evaluation of the effect of the *SLC22A12* variation on serum urate levels

Uric acid (UA) level can be modeled as the sum of genetic and environmental effects:

U=G+E. (1)

The phenotypic variance in the trait V(P) is the sum of the effects as follows:

V(U)=V(G)+V(E)+Cov(G,E). (2)

In this paper, we assume the covariance of environmental and genetic factors, Cov(G,E), to be 0. Genetic effect is the sum of additive and nonadditive genetic effects. The broad sense heritability, $H^{2}$, is defined as

$$H^{2}=\frac{V\left(G\right)}{V\left(U\right)}$$ (3)

To examine the effects of *SLC22A12* variants on serum urate levels, multivariable modeling was performed after adjusting for potential covariates: age at examination, BMI, and presence of diabetes mellitus. In this study, the environmental factors were divided into two factors:

$$E=E_{\text{known factors}}+E_{\text{unknown factors}}.$$ (4)

By using the additivity of the variance,

$$V\left(E\right)=V\left(E_{\text{known factors}}\right)+V\left(E_{\text{unknown factors}}\right).$$ (5)

Multivariable modeling was performed after adjusting for potential covariates: age at examination, eGFR, BMI, and HbA1c. We analyzed male and female samples separately. By using this model, the serum urate variable was adjusted to be as unaffected by environmental factors as possible:

$$V\left(U_{adjusted}\right)=V\left(U\right)-V(E_{\text{known factors}}).$$ (6)

By using the adjusted serum urate level, we aimed to identify additional associated genetic variants.

The difference in serum urate levels between the samples of men and women was tested by *t*-test. BMI is weight in kilograms divided by the square of height in meters. These values were used in multiple regression analyses performed by the “glm” function using the statistical software R 3.5.1 (R Core Team 2018).

eGFR was calculated by the corresponding formula for each participant (Inker *et al.* 2012). Then, a factor of 0.908 for Japanese individuals was multiplied with the resultant values (Horio *et al.* 2013).

### Estimation of variance explained SNPs of the *SLC22A12* gene

We denoted the additive and nonadditive genetic effects as *A* and *D*, respectively.

G=A+D (7)

Nonadditive genetic effect includes dominant, epistatic, maternal, and paternal effects. V(A) is the variance due to the additive genetic effects. The additive genetic portion of the phenotypic variance is known as narrow-sense heritability and is defined as

$$h^{2}=\frac{V\left(A\right)}{V\left(U\right)}$$ (8)

In this study, we estimated the additive genetic effect of the variants on *SLC22A12* gene:

$$A=A_{SLC22A12}+A_{\text{Other genetic factors}}.$$ (9)

The additive effect of *SLC22A12* variants on the variance of serum urate levels was estimated by additivity of the variance. The variance can be divided into two factors:

$$V\left(A\right)=V\left(A_{SLC22A12}\right)+V\left(A_{\text{Other genetic factors}}\right).$$ (10)

By denoting$V_{1}\left(U_{\text{adjusted}}\right)$ and $V_{2}\left(U_{\text{adjusted}}\right)$ as the variances of serum urate levels of the entire group (homozygotes of the wild-type *SLC22A12*) and subjects that had *SLC22A12* variants, respectively, and $n_{1}$ and $n_{2}$ as the number of subjects in the wild-type SLC22A12 homozygotes and subjects that have *SLC22A12* variants, respectively,

$$V\left(U_{\text{adjusted}}\right)=\frac{n_{1}V_{1}\left(U_{\text{adjusted}}\right)+n_{2}V_{2}\left(U_{\text{adjusted}}\right)}{n_{1}+n_{2}}+V\left(A_{SLC22A12}\right).$$

The proportion of variance explained by SNPs of *SLC22A12* can be estimated as follows:

$$h_{SLC22A12}^{2}=\frac{V\left(A_{SLC22A12}\right)}{V\left(U\right)}.$$ (11)

### Burden test

We grouped the subjects according to whether or not they contained the variant that affected urate levels strongly. All included variants were assumed to be independent. Differences between mean serum urate levels in the wild-type and that of variants were compared using the *t*-test. This test is equivalent to a burden test (Lee *et al.* 2014) with the same weight for all variants.

### Data availability

The authors state that all data necessary for confirming the conclusions presented in the article are represented fully within the article. Integration of health and genome data involves privacy violation risks. To protect privacy of the cohort participants, the TMM has established a policy of data sharing based on the policies of HIPAA, NIH, and Sanger Institute. Request for the use of the TMM biobank data for research purposes should be made by applying to the ToMMo headquarters. All requests are subject to approval by the Sample and Data Access Committee. Details are available upon request at dist@megabank.tohoku.ac.jp.

The request will be reviewed by the following criteria: (1) urgency, (2) scientific validity, (3) feasibility, (4) contribution to human health including the cohort participants, and (5) security of the requesting organization. The data are anonymized before they are provided. These criteria are shown on the TMM website:

https://www.megabank.tohoku.ac.jp/tommo/qa/all-megabank.

The Sample and Data Access Committee is organized by the ToMMo and the IMM. The Sample and Data Access Committee consists of specialists from a wide variety of research fields, including human genetics, epidemiology, and jurisprudence. The chairperson and the majority of the members of the committee do not belong to Tohoku University or Iwate Medical University, which undertakes TMM projects.

The committee evaluates reidentification risks of individual datasets. When the risk is very high, the data cannot be accessed (entire genome is an example of “very high risk” data). When the risk is high, data will be shared in a specific network. When the risk is standard, the data can be transferred. Computer programs, DNA sequences, experimental protocols, and antibodies are available from the authors on request. Details are described in Takai-Igarashi *et al.* (2017).

## Results

### Variants of the *SLC22A12* gene found by whole-genome sequencing

On the coding region of SLC22A12, 8 synonymous sites and 14 nonsynonymous sites segregated among 1285 participants. The rs IDs of synonymous sites are rs148378818, rs571307205, rs3825017, rs3825016, rs11231825, rs1272829728, rs7932775, and rs200072517. Nonsynonymous variations are listed in Table 1. The nonsynonymous sites consist of 11 missense sites, two stop-gain sites, and a splice site. We call SNPs that are not registered in dbSNP Build 151 novel SNPs. Novel SNPs (indicated in Table 1) show alternative allele frequency (%)found on the SLC22A12 from 1285 participants of ToMMo cohort study. Minor alleles found in this study were not found in 1000G samples, except in East Asia (Table 1). Only one individual was compound heterozygous. Table 1 shows nonsynonymous variations of *SLC22A12* that were reported in previous studies in italic letters. All nonsynonymous variations except for those found in the Japanese (Ichida *et al.* 2004; Sakiyama *et al.* 2016) and Korean population (Lee *et al.* 2008; Cho *et al.* 2015) were found in 2KJPN.

**Table 1:** SNPs and minor allele frequency (%) that were found on the *SLC22A12* from 1285 participant of ToMMo cohort study

Position		SNP ID	Amino acid change	Reference allele	Alternative allele	2KJPN		1000G				Reference
						Men	Women	JPT	CHB	CHS	Other	
64359297		rs121907896	p.Arg90His	G	A	0.95	0.93	0.00	0.00	0.00	0.00	Cho *et al.* (2015)
												Ichida *et al.* (2004)
												Lee *et al.* (2008)
												Sakiyama *et al.* (2016)
*64359302*		*rs144328876*	*p.Arg92Cys*	C	T	*0.00*	*0.00*	*0.00*	*0.00*	*0.00*	*0.00*	Graessler *et al.* (2006)
												Mancikova *et al.* (2016)
64359320		rs930110938	p.Leu98Phe	C	T	0.16	0.00	0.00	0.00	0.00	0.00	
64360355		rs58174038	c. 506+1G	G	A	0.16	0.31	0.48	0.00	0.00	0.00	
64360873		rs144325235	c.507-4G > A	G	A	0.16	0.00	0.00	0.00	0.00	0.00	
*64360977*		*rs374743769*	*p.Arg203Cys*	*C*	*T*	*0.00*	*0.00*	*0.00*	*0.00*	*0.00*	*0.00*	Mancikova *et al.* (2016)
64360996		rs552232030	p.Ala209Val	C	T	0.00	0.15	0.00	0.49	0.00	0.00	
64361020		rs121907893	p.Thr217Met	C	T	0.00	0.15	0.00	0.00	0.00	0.00	Enomoto *et al.* (2002)
64361122		rs145738825	p.Ala226Val	C	T	0.16	0.00	0.00	0.00	0.00	0.00	
64361124		rs201136391	p.Ala227Thr	G	A	0.16	0.00	0.48	0.00	0.48	0.00	
64361219		rs121907892	p.Trp258Ter	G	A	4.60	3.71	2.40	0.00	0.00	0.00	Enomoto *et al.* (2002)
												Ichida *et al.* (2004)
												Lee *et al.* (2008)
												Sakiyama *et al.* (2016)
64366046		Novel	p.Gln297Ter	C	T	0.16	0.00	0.00	0.00	0.00	0.00	
64366080		Novel	p.Lys308Arg	A	G	0.00	0.15	0.00	0.00	0.00	0.00	
*64366298*		*rs150255373*	*p.Arg325Trp*	C	T	*0.00*	*0.00*	*0.00*	*0.00*	*0.00*	*0.02*	Tin *et al.* (2018)
*64367290*		*rs563239942*	*p.Arg405Cys*	C	T	*0.00*	*0.00*	*0.00*	*0.00*	*0.00*	*0.02*	Tin *et al.* (2018)
*64367322*			*p.Leu415_Gly417del*	GGCAGGGCT	G	*0.00*	*0.00*	*0.00*	*0.00*	*0.00*	*0.00*	Claverie-Martin *et al.* (2018)
*64368212*		*rs200104135*	*p.Thr467Met*	C	T	*0.00*	*0.00*	*0.00*	*0.00*	*0.00*	*0.21*	Claverie-Martin *et al.* (2018)
												Tin *et al.* (2018)
												Vidanapathirana *et al.* (2018)
64368218		Novel	p.Val469Ala	T	C	0.16	0.00	0.00	0.00	0.00	0.00	
*64368239*		*rs148862453*	*p.Ala476Asp*	C	A	*0.00*	*0.00*	*0.00*	*0.00*	*0.00*	*0.00*	Claverie-Martin *et al.* (2018)
64368242		rs773677616	p.Arg477His	G	A	0.00	0.31	0.00	0.00	0.00	0.00	Iwai *et al.* (2004)
												Stiburkova *et al.* (2013)
												Stiburkova *et al.* (2013)
64368409		rs1382724038	p.Gln533Lys	C	A	0.00	0.00	0.00	0.00	0.00	0.00	
*64368968*		*rs528619562*	*p.Lys536Thr*	A	C	*0.00*	*0.00*	*0.00*	*0.00*	*0.00*	*0.02*	Tin *et al.* (2018)
64368979		Novel	p.His540Tyr	C	T	0.00	0.15	0.00	0.00	0.00	0.00	

Amino acid changes are based on ENST00000377574.

**Figure 1:**
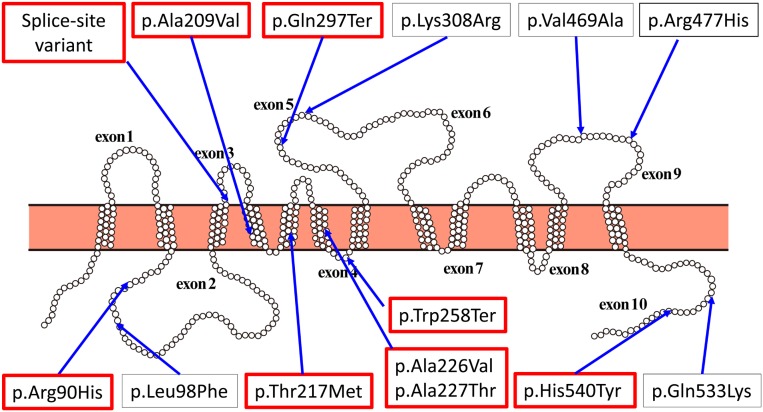
Topology of URAT1 encoded by *SLC22A12* with positions of nonsynonymous variants indicated. Amino acid changes with strong effects on urate uptake are indicated by red boxes.

The structure of URAT1 and amino acid changes of URAT1 are shown in Figure 1. Amino acid sequences are based on ENST00000377574. MAF of 2KJPN, JPT, CHB, CHS, and other populations in the 1000G project are shown in Table 1. The effects of four sites among 11 missense variants and two stop-gain variants have already been investigated (Enomoto *et al.* 2002; Ichida *et al.* 2004; Iwai *et al.* 2004). Thus, we assessed urate uptake by eight nonsynonymous variants. We also investigated one splice site variant.

### URAT1 expression in *Xenopus* oocyte detected by immunofluorescence

**Figure 2:**
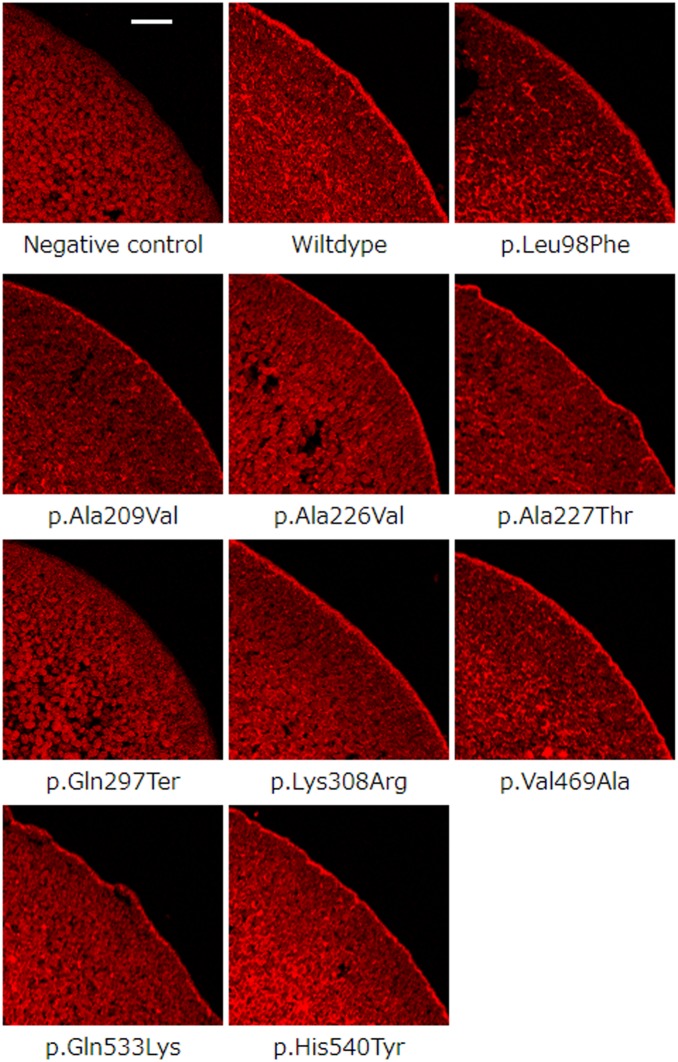
Subcellular localization of the URAT1 of the wildtype and variants of the *SLC22A12* cRNA in *Xenopus* oocytes. Bar, 100 µm. The URAT1 proteins except those injected p.Gln297Ter and p.Gln297Ter complementary RNA (cRNA) are expressed at the plasma membrane, whereas fluorescence levels were undetectable in oocytes injected with water or those injected with p.Gln297Ter, p.Gln297Ter complementary RNA (cRNA).

Subcellular localization in oocytes of the wild-type or variant URAT1 was detected by immunostaining with a specific antibody raised against the fourth extracellular loop flanked by transmembrane domains 7 and 8 of URAT1 (Figure 2). Figure 2 shows subcellular localization of the wild type and mutants in oocytes. Immunodetection with a specific antibody raised against the C terminus of URAT1 showed that the wild-type, p.Leu98Phe, p.Ala209Val, p.Ala226Val, p.Ala227Thr, p.Lys308Arg, p.Val469Ala, p.Gln533Lys, and p.His540Tyr proteins are expressed at the plasma membrane. On the contrary, fluorescence levels were undetectable in oocytes injected with water or those injected with p.Gln297Ter cRNA.

### Effects of amino acid substitution on urate uptake by URAT1

Table 2 shows the effects of amino acid substitution on urate uptake by URAT1. p.Leu98Phe, p.Lys308Arg, p.Val469Ala, or p.Gln533Lys did not decrease urate uptake. However, p.Ala209Val, p.Ala226Val, p.Ala227Thr, p.Gln297Ter, or p.His540Tyr significantly reduced urate uptake (Welch two-sample *t*-test, *P* < 0.01). By using *Xenopus* oocytes, previous studies have shown that p.Arg90His, p.Thr217Met, p.Trp258Ter, or p.Arg477His affect serum urate levels (Enomoto *et al.* 2002; Ichida *et al.* 2004; Iwai *et al.* 2004); thus, nine variants were examined in this study. Amino acid changes with strong effects on urate uptake are indicated by red boxes in Figure 1.

**Table 2:** Effects of amino acid substitution on urate uptake by URAT1 (pmol/oocyte/hour)

SNP ID	Amino acid change	Mean	SE	*P*	Significance
Wildtype	No Change	8.202	0.412	1.00	
rs930110938	p.Leu98Phe	8.275	0.205	0.88	
rs552232030	p.Ala209Val	0.215	0.073	0.00	^a^
rs145738825	p.Ala226Val	5.659	0.181	0.00	^a^
rs201136391	p.Ala227Thr	3.830	0.291	0.00	^a^
chr11:64366046	p.Gln297Ter	0.115	0.058	0.00	^a^
chr11:64366080	p.Lys308Arg	8.960	0.469	0.24	
chr11:64368218	p.Val469Ala	8.720	0.654	0.51	
rs1382724038	p.Gln533Lys	7.898	0.298	0.56	
chr11:64368979	p.His540Tyr	0.175	0.039	0.00	^a^
Negative control	Water	0.075	0.027	0.00	^a^

Amino acid changes are based on ENST00000377574.

a Urate uptake is significantly lower than wildtype.

### Effect of variation on splicing

**Figure 3:**
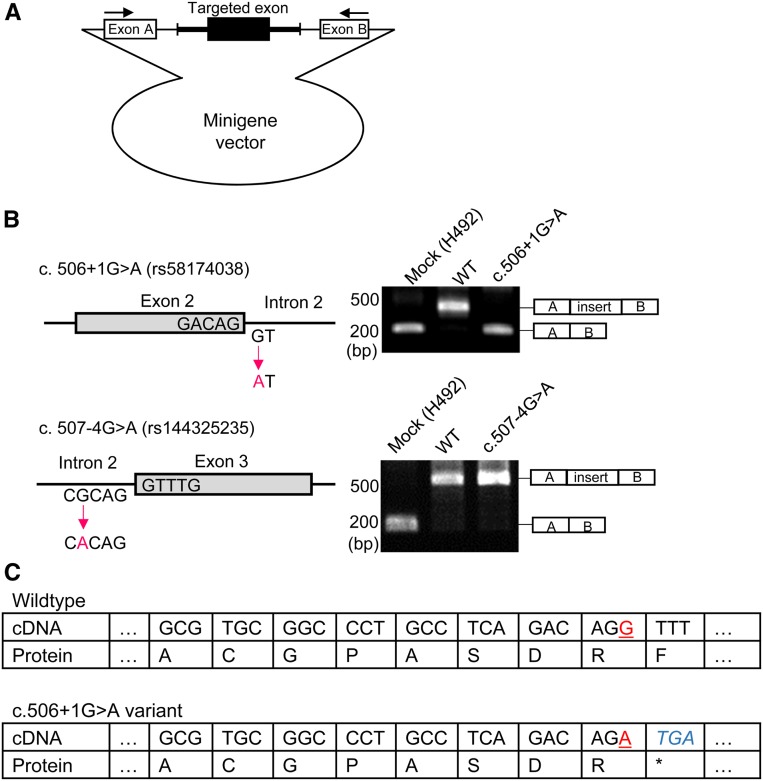
Effects of variant mutations on splicing. (A) Diagram of the minigene. (B) Effect of variants on splicing. rs58174038 (c. 506+1G > A) caused aberrant splicing, whereas rs144325235 did not affect splicing. (C) Putative effect of the splice-site variation rs58174038 (c. 506+1G > A) of the *SLC22A12* gene on URAT1. Two alleles of rs58174038 are indicated by underlined red letters. Genomic sequences are indicated by italic blue letters. This figure indicates that A allele will cause loss-of-function of URAT1 because of the stop codon TGA located in the genomic sequence.

We also found two variants close to the exon–intron boundary, rs58174038 (c. 506+1G > A) and rs144325235 (c. 507-4G > A), located on the junction between exon 2 and intron 2, and intron 2 and exon 3 of *SLC22A12*, respectively (Figure 1). Because variants in exon–intron boundaries can affect the splicing location, and result in abnormal transcripts by aberrant exon skipping (Takeuchi *et al.* 2015), we evaluated the influence of these two variants on the splicing pattern of the transcripts by *in vitro* splicing assay using a hybrid minigene (Figure 3A). The assay showed that the c. 506+1G > A variant caused aberrant exon skipping to exclude exon 2 (Figure 3B). In contrast, the c. 507-4G > A variant did not affect the splicing pattern. These data suggest that rs58174038 (c. 506+1G > A) causes aberrant splicing, while rs144325235 does not. Figure 3C shows the genomic sequences around rs58174038. The sequence indicates that the splice variant c. 506+1G > A presumably has a stop codon that would cause loss of function of URAT1. This site is also indicated by a red box in Figure 1.

### Evaluation of the effect of *SLC22A12* variation on serum urate levels

Table 3 summarizes the serum urate levels and characteristics of other covariates of the study subjects. The serum urate levels are reported in mg/dl in Table 3 to compare the serum urate levels with previous studies (Krishnan *et al.* 2012; Major *et al.* 2018b). These values can be converted to μmol/l if multiplied by 59.48 (Panoulas *et al.* 2008). The average serum urate level of the samples obtained from men was 6.07 mg/dl, which differed significantly from that of the samples obtained from women, which was 4.57 mg/dl (*t*-test, *P* < 10^−22^). Table 3 also summarizes mean values and SD for age, eGFR, BMI, and HbA1c levels of the subjects, and presents the linear-regression results. The eGFR levels explain about 10% of the variance in serum urate level. The effects of BMI or HbA1c were much smaller than that of eGFR.

**Table 3:** Characteristics of the study subjects (*n* = 1,285) and linear regression on serum uric acid and covariates

Variable	Mean	SD	β	SE	*P*	Significance
Men (*n* = 631)						
Serum uric acid (mg/dL)	6.07	1.31				
Age (year)	61.61	11.23	0.0319	0.0053	2.29×10^−9^	^a^
Body mass index (kg/m^2^)	24.31	3.17	0.0635	0.0150	4.27×10^−5^	^a^
eGFR (mL/minute/1.73m^2^)	90.77	13.65	−0.0368	0.0043	2.00×10^−16^	^a^
HbA1c	5.46	0.71	−0.2289	0.0690	0.000957	^a^
Women (*n* = 647)						
Serum uric acid (mg/dL)	4.57	0.96				
Age (year)	57.26	13.17	−0.0192	0.0038	5.99×10^−7^	^a^
BMI (kg/m2)	23.08	3.65	0.0592	0.0099	3.12×10^−9^	^a^
eGFR (mL/minute/1.73m^2^)	96.09	14.39	−0.0297	0.0034	2.00×10^−16^	^a^
HbA1c	5.34	0.36	0.2652	0.1067	0.0132	

eGFR, estimated glomerular filtration rate; SD, standard deviation; SE, sampling error.

a Significantly correlated.

**Figure 4:**
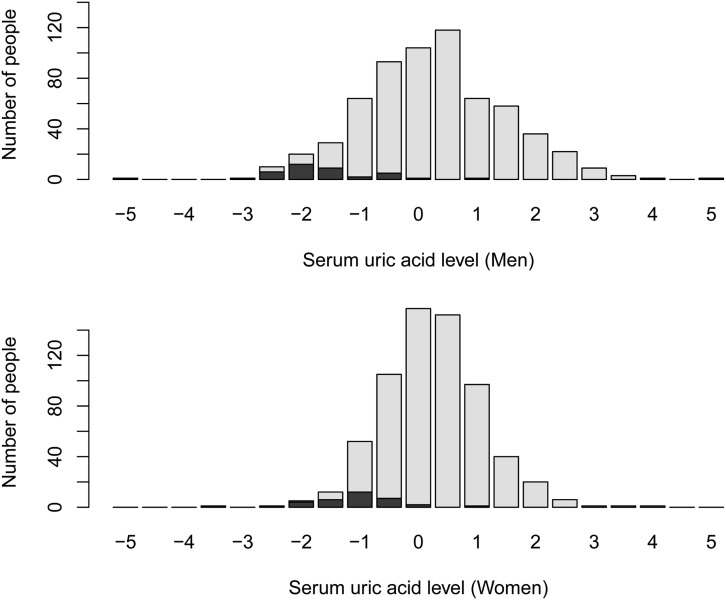
Distributions of adjusted serum urate levels. The *X*-axis indicates the serum urate level, while the *Y*-axis represents the participants’ number. Gray boxes represent those with the wild-type URAT1; closed boxes represent those with variants.

Figure 4 shows the distribution of adjusted serum urate levels. The Kolmogorov–Smirnov test did not reject normality. We grouped the participants with variations indicated by red boxes in Figure 1. In Figure 4, black boxes indicate participants with variants. It is worth noting that variations indicated by red boxes in Figure 1 are damaging urate reabsorption, so that serum urate levels of individuals with variants of the *SLC22A12* gene are lower than those with wild type. Table 4 summarizes the differences in adjusted serum urate levels in participants with variations indicated by red boxes in Figure 1 and wild-type carriers. The difference was significant (*t*-test, *P* < 10^−16^).

**Table 4:** The difference in adjusted serum uric acid level between carriers of variants and that of wildtype

	Number of participants	Mean	SD	*P*^a^
Men				
All	634	0.00	1.25	
Variants	38	−1.92	0.87	< 10^−16^
WT	596	0.12	1.25	
Women				
All	651	0.00	0.89	
Variants	34	−1.34	0.66	< 10^−16^
WT	617	0.07	0.62	

a Significantly different from adjusted serum urate level of wild-type carriers (t-test, *P* < 10^−16^).

Table 5 presents a summary of variances explained by environmental and genetic factors. The number of participants with variations indicated by red boxes in Figure 1 is also shown in Table 5. The variances are shown in (mg/dl)^2^. These values can be converted to (μmol/l)^2^ if multiplied by (59.48)^2^. By using the SD presented in Table 4, the variances of serum urate levels could be explained by *SLC22A12* variants, which were 0.23 and 0.10 for men and women, respectively. Thus, *h*^2^_URAT1_ was 13.3% and 10.5% for men and women, respectively (Table 5). Table 5 also shows that age at examination, eGFR, BMI, and HbA1c explain about 10% of serum urate levels.

**Table 5:** Summary of variances explained by environmental and genetic factors

	Variances	Portion (%)
Men		
*V* (*U*)	1.73	100
*V* (*E*_known factors_)	0.16	9.4
*V* (*A _SLC22A12_*)	0.23	13.3
Women		
*V* (*U*)	0.92	100
*V* (*E*_known factors_)	0.13	13.7
*V* (*A _SLC22A12_*)	0.10	10.5

*V* (*E*_known factors_), Variance explained by known environmental factors; *V* (*A _SLC22A12_*), Additive variance explained by genetic variations of the. SLC22A12 gene.

## Discussion

To discover genetic variants that explain the missing heritability of serum urate levels, we examined the *SLC22A12* variation in the ToMMo cohort. As a result, we detected 13 nonsynonymous sites segregated among 1285 participants (Table 1). Among these sites, previous studies have shown that four nonsynonymous sites affect urate uptake by URAT1, which is encoded by *SLC22A12*. We assessed urate uptake by nine variants, and showed that four variants sharply reduced the URAT1 function (Figure 1 and Table 2). We also conducted minigene experiments and found that one variation caused abnormal splicing in the *SLC22A12* transcript (Figure 3). We grouped the participants with variants that affected urate uptake by URAT1, and analyzed variance in serum urate levels. The results showed that the *h*^2^_URAT1_ values for men and women are >10%, suggesting that rare variants underlie a substantial portion of the “missing heritability” of serum urate levels (Table 5).

Urate-uptake experiments showed that the effects of amino acid substitutions on protein function depended on substitution location in the protein primary structure (Figure 1 and Table 2). p.Gln297Ter, p.Trp258Ter, and slice-site variant truncated proteins presumably have a stop codon that would cause loss of function of URAT1. Substitutions in the transmembrane domain, p.Ala209Val, p.Thr217Met, p.Ala226Val, and p.Ala227Thr strongly affect the transporter activity of URAT1. On the other hand, the missense variations on the extracellular loops of the URAT1 protein, p.Lys308Arg, p.Val469Ala, and p.Arg477His have smaller effects on the transporter activity of URAT1 than do other variants. Substitution experiments showed that the effect of p.Ala209Val was significantly stronger than that of p.Ala226Val (*P* = 3.52 × 10^−12^; Welch two sample *t*-test), although both substitutions featured Ala–Val exchange. These findings provide new insights into the URAT1 structure–function relationships. Tin *et al.* (2018) showed that a higher CADD Phred score (Rentzsch *et al.* 2019) was associated with substantial adverse effects on serum urate among variants. In this study, we did not find any significant relationship between CADD Phred score and functional change of URAT1, likely because of a small number of loci (data not shown).

Although urate levels are known to be affected by various environmental factors (Major *et al.* 2018b), Table 3 indicates that serum urate levels are influenced primarily by eGFR. UA is freely filtered at the glomerulus, with the majority undergoing reabsorption via proximal tubular urate transporter proteins, and about one-tenth being secreted back into the filtrate in the late proximal tubules (Tasic *et al.* 2011; Iseki *et al.* 2013). Our results support the importance of kidney function in regulating serum urate levels.

Purine metabolic disorders, such as a reduced activity of hypoxanthine-guanine phosphoribosyl-transferase and overactivity of phosphoribosyl pyrophosphate synthetase, are associated with serum urate level (Nyhan 2005). However, patients suffering from purine metabolic disorders associated with pathological concentrations of serum uric acid (SUA) were not excluded, because the questionnaire to check for metabolic disorders of purine was not presented to cohort participants.

Table 2 also indicates that serum urate levels are also influenced by glucose level. This result is consistent with previous studies showing that SUA levels decrease in diabetes patients who have received an inhibitor of sodium-glucose cotransporter 2 (SGLT2) (Davies *et al.* 2015; van Bommel *et al.* 2017; Ouchi *et al.* 2018). A previous cohort study showed that serum urate levels increased with moderately increased levels of HbA1c, and then decreased as levels of HbA1c increased further (a U-shaped relationship) (Cook *et al.* 1986; Choi and Ford 2008). Therefore, a nonlinear model might be necessary.

A previous study showed that BMI was significantly associated with serum urate levels, even after adjusting for genetic, family, and environmental factors in both men and women (Tanaka *et al.* 2015). Serum urate levels in men are known to be much higher than those in women (Fang and Alderman 2000). The multivariate analyses in this study were consistent with the previous findings.

The variance of the adjusted urate levels includes the variances driven by unadjusted environmental factors, such as diet. Diet has been identified as a risk factor for the development of gout (Tsai *et al.* 2012; Dalbeth *et al.* 2016). Higher levels of meat and seafood consumption are associated with higher levels of SUA (Choi *et al.* 2005). Coffee consumption is associated with lower SUA level (Choi and Curhan 2007). A recent study (Major *et al.* 2018b) showed that diet explains minimal variation in serum urate levels in the general population. In this study, we did not incorporate the effect of food and drinks on the serum urate level, because the answers from the participants of the TMM cohort study have missing data. In future, diet effects on serum uric level will be incorporated.

Our results suggest that rare functional SNPs are major contributors to missing heritability. Including rare SNPs in genotyping platforms will advance identification of causal SNPs (Gorlov *et al.* 2008). Minor alleles found in this study were not found in 1000G samples, except in East Asia (Table 1). The rare variants of *SLC22A12* have strong ethnic specificity. In addition, there must be rare variants of many genes other than the SLC22A12 gene that affect SUA. Rare variants on ABCG2 were identified in a Czech population (Stiburkova *et al.* 2017). In addition to common and rare variants, epigenetic factors are expected to explain the “missing heritability” (Manolio *et al.* 2009; Eichler *et al.* 2010), and whole-genome bisulfite sequencing of TMM cohort samples is ongoing (Hachiya *et al.* 2017).
